# Ceramide releases exosomes with a specific miRNA signature for cell differentiation

**DOI:** 10.1038/s41598-023-38011-1

**Published:** 2023-07-07

**Authors:** Federico Fiorani, Rossana Domenis, Emiliano Dalla, Samuela Cataldi, Carmela Conte, Martina Mandarano, Angelo Sidoni, Adriana Cifù, Tommaso Beccari, Alessandra Mirarchi, Cataldo Arcuri, Francesco Curcio, Elisabetta Albi

**Affiliations:** 1 Department of Pharmaceutical Sciences, University of Perugia, 06126 Perugia, Italy; 2 Department of Medicine (DAME), University of Udine, 33100 Udine, Italy; 3 Division of Pathological Anatomy and Histology, Department of Medicine and Surgery, University of Perugia, 06126 Perugia, Italy; 4 Department of Medicine and Surgery, University of Perugia, 06126 Perugia, Italy

**Keywords:** Cell biology, Developmental biology, Neuroscience

## Abstract

Exosomes are well established effectors of cell–cell communication. Their role on maturation of embryonic cells located in hippocampus, seat of memory, is unknown. Here we show that ceramide facilitates release of exosomes from HN9.10e cells extending information for cell differentiation to neighboring cells. We found only 38 miRNAs differentially expressed in exosomes derived from ceramide-treated cells in comparison with control cells (including 10 up-regulated and 28 down-regulated). Some overexpressed miRNAs (mmu-let-7f-1-3p, mmu-let-7a-1-3p, mmu-let-7b-3p, mmu-let-7b-5p, mmu-miR-330-3p) regulate genes encoding for protein involved in biological, homeostatic, biosynthetic and small molecule metabolic processes, embryo development and cell differentiation, all phenomena relevant for HN9.10e cell differentiation. Notably, the overexpressed mmu-let-7b-5p miRNA appears to be important for our study based on its ability to regulate thirty-five gene targets involved in many processes including sphingolipid metabolism, sphingolipid-related stimulation of cellular functions and neuronal development. Furthermore, we showed that by incubating embryonic cells with exosomes released under ceramide treatment, some cells acquired an astrocytic phenotype and others a neuronal phenotype. We anticipate our study to be a start point for innovative therapeutic strategies to regulate the release of exosomes useful to stimulate delayed brain development in the newborn and to improve the cognitive decline in neurodegenerative disorders.

## Introduction

Ceramides (Cers) are a class of molecules that contain an amide group in the sphingoid base of 18 carbons that acts as a skeleton to which a fatty acyl chain of variable length is bound^1^. Cers are central intermediates of sphingolipid (Sph) metabolism. They can be generated by (i) a de novo synthetic pathway, (ii) the degradation of sphingomyelin (SM) and/or complex sphingolipids as glucocerebrosides, (iii) the salvage pathway in which sphingosine derived from ceramide can be reacylated or (iv) the reverse action of ceramidases (Cerase)^2^. Since Cers are highly hydrophobic, they are molecules poorly water-soluble and tend to increase the molecular order in membranes as plasma membrane, nuclear and mitochondrial envelope, endoplasmic reticulum and Golgi apparatus^3^. Alternatively, Cers are transported to the trans Golgi network by ceramide transfer protein (CERT) or by vesicular traffic for SM biosynthesis^4^. The length of the fatty acyl chain (from 14 to 26 carbons) is a critical factor in the molecular actions of the Cers^5^. The C18 acyl chains most strongly stimulate exosome release from neuronal cells^6^. C16-C22 Cers were found to be positively associated with type-2 diabetes mellitus incidence^7^. C16 Cer and C22:2 dihydroceramide were associated with higher risk of cardiovascular diseases^8^.

In laboratory, short-chain cell-permeable Cer analogues (C2-ceramide and C6-ceramide) have been extensively used because they are more efficient at passing through the plasma membrane than Cer with a longer acyl chain^9–11^. Cers produced from SM by sphingomyelinases (SMases) exert anti-proliferative and pro-apoptotic^12^ activities in cells. However, many studies showed that they are able to induce cell differentiation^13^, also in embryonic stem cells^14,15^. The acid SMase (aSMase) encoded by SMPD1 is involved in apoptosis signaling and cancer^16^, neutral SMase1 (nSMase1) encoded by SMPD2 is activated in response to stress, inflammation, radiation, and cancer^17–19^, nSMase2 encoded by SMPD3 plays a role in cell growth arrest, inflammatory response and cancer^1,7,20^, nSMase3 encoded by the SMPD4 gene is involved in keratinocyte proliferation and differentiation^21^ and in embryonic hippocampal cell differentiation stimulated by vitamin D3 treatment^22^ with consequent release of exosomes^23^.

Exosomes are small extracellular vesicles (30–120 nm in diameter), experienced to be in three stages such as EE (early endosome), ILE (intraluminal vesicle), and MVBs (multivesicular bodies), that are released from cells in response to specific stimuli under physiological or pathological conditions. They are mainly composed by proteins, lipids, small amounts of DNA and mRNA, and transfer specific molecular cargoes, as non-coding RNAs that mainly include microRNAs (miRNAs), long noncoding RNAs, circular RNAs, PIWI-interacting RNAs, and small interfering RNA^24^. Specifically, miRNAs are small 20–23 nucleotides non-coding RNA^25^. It has been highlighted that exosomal miRNAs can regulate many cellular processes i.e. cell proliferation, migration, apoptosis, autophagy and differentiation by modulating the gene expression in target cells^26^. Thus, exosomes are considered as extracellular messengers in the microenvironment by influencing cell function^27^. Of particular interest is the role of exosomes in the proliferation, differentiation and/or apoptosis of neuronal cells. Of note, they are able to transmit functional genetic information by mediating communication between different cell types in the brain^27^. Thus, exosomes can influence the hallmarks of neuronal cells^28^ and, consequently, the pathophysiology of the central nervous system^29^.

Mounting evidence has shown the role of exosomes in neurodegenerative processes and in tumors of the central nervous system^27^. The role of exosomes in the differentiation of embryonic/stem cells of the nervous system is on the contrary underinvestigated.

As mentioned above, lipids are essential molecules of exosomal membranes. Specific lipids are known to be enriched in exosomes relative to their parent cells, especially Sphs^30^. In the nervous system, a dysregulation of Sph induces abnormalities in exosomes resulting in the dysfunctions in inter-neuronal communication and in diseases promotion^31^.

The degradation of the SM present in cell membrane by nSMase action is responsible for the increase in membrane fluidity (both for the reduction of the SM and for the release of the cholesterol bound to it) and for the production of Cer which, having a cone-shaped structure, can confer a spontaneous curvature to the membrane with release of exosomes^32^. In fact, the authors report that exosome release is markedly reduced with nSMase inhibitors, although not in all cells. Additionally, Trajkovic et al.^33^ demonstrated that endosome-exosome binding is not dependent on ESCRT (endosome sorting complex required for transport), but requires Cer. Thus, Cer plays an essential role in the functioning of exosomes in different pathophysiological conditions. In the brain, exosomal Cer contributes to the diffusion of neurotoxic proteins or their clearance^34^.

We have previously shown that vitamin D3 induces embryonic hippocampal cell differentiation (HN9.10e cell line) via both increase of nSMase activity and upregulation of the nSMase protein^35^. As a consequence, exosomes enriched in Cer are released^23^. Incubation of the cells with the same Cer concentration of exosomes is necessary and sufficient to stimulate the same HN9.10e cell differentiation obtained with vitamin D3^23^.

Therefore, we set out to determine whether the progression over time of the differentiating effect of Cer is mediated by the miRNAs present in the exosomes released after treatment. In this way, exosomes could be an explanation for the propagation of the differentiating effect in brain embryonic cells after an early and single administration of Cer.

## Results

### Effect of ceramide on HN9.10e cells

**Figure 1:**
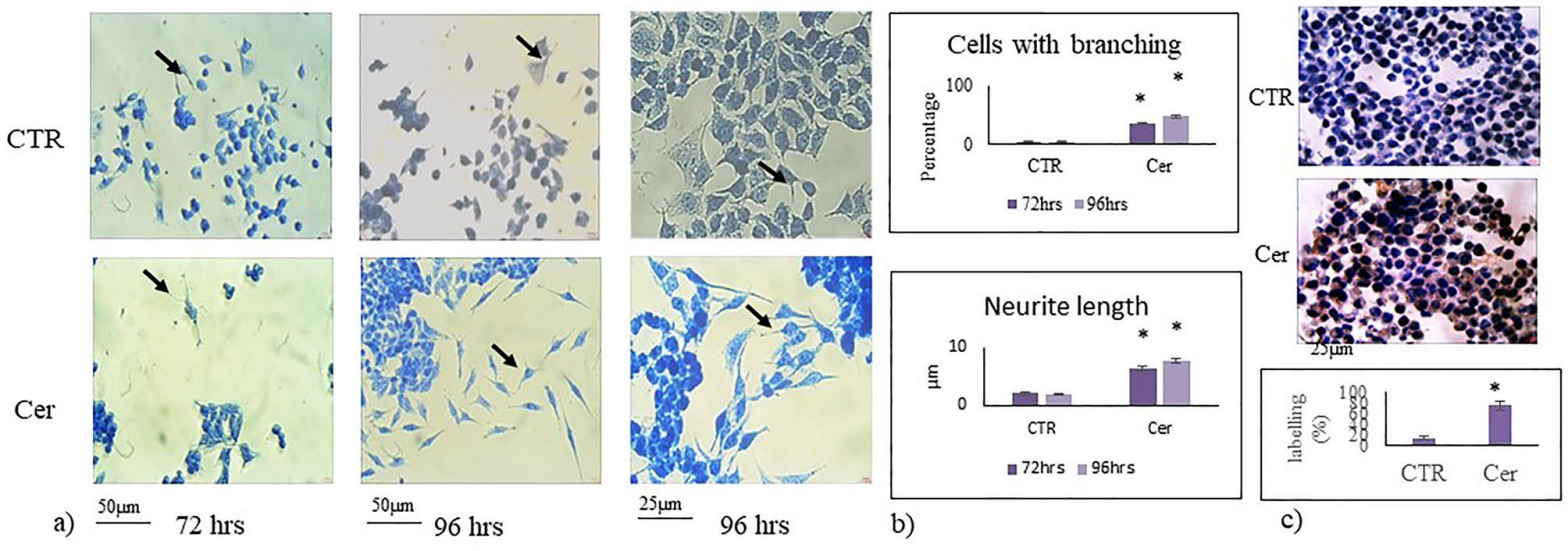
Effect of ceramide on HN9.10e cells. HN9.10e cells were cultured without (CTR) or with 100 nM Ceramide (Cer). (**a**) morphologic analysis after 72 and 96 h of cell culture. The observations were performed by using inverted microscopy EUROMEX FE 2935 (ED Amhem, Netherlands) equipped with a CMEX 5000 camera system; on the left and in the center 20** × **magnification, on the right 40** × **magnification. Arrows point to differentiated cells with soma modification and the presence of neurites. (**b**) The morphometric analysis was performed by using ImageFocus software.c) immunocytochemical analysis of Neurofilament 200 kDa (NF200) expression in HN9.10e cells. The observations were performed as above reported. Above pictures (40 × magnification) and below the percentage of total cells stained in brown (positive cells). Data were expressed as mean ± SD of three independent experiments performed in duplicate. Significance versus the control sample, *p < 0.01.

Cer was previously shown to quickly induce HN9.10e cell differentiation when used at the same concentration present in exosomes released under Vitamin D3 treatment^23^. In order to determine whether HN9.10e cells proceed to differentiation without further administration of Cer, assuming a possible propagation of the process via cell–cell communication, we evaluated the cell morphology and neurofilament protein (NF200) expression. The results showed robust effects of Cer treatment as time progressed; this was observed by comparing data at 96 h with those obtained at 72 h (Fig. 1a,b). The morphological differentiation was characterized by the change of the soma shape and appearance of neurites^22,23^. In control sample, although the shape of the soma changed from 72 to 96 h (Fig. 1a), the number of the cells with branching and the length of the neurites remained constant, always with low values (Fig. 1b). The Cer treatment strongly changed the cell morphology in time (Fig. 1a). Interestingly, the number of branching cells and the length of the neurites increased (Fig. 1b). In support, the expression of NF200 significantly increased in the Cer treated sample (Fig. 1c).

**Figure 2:**
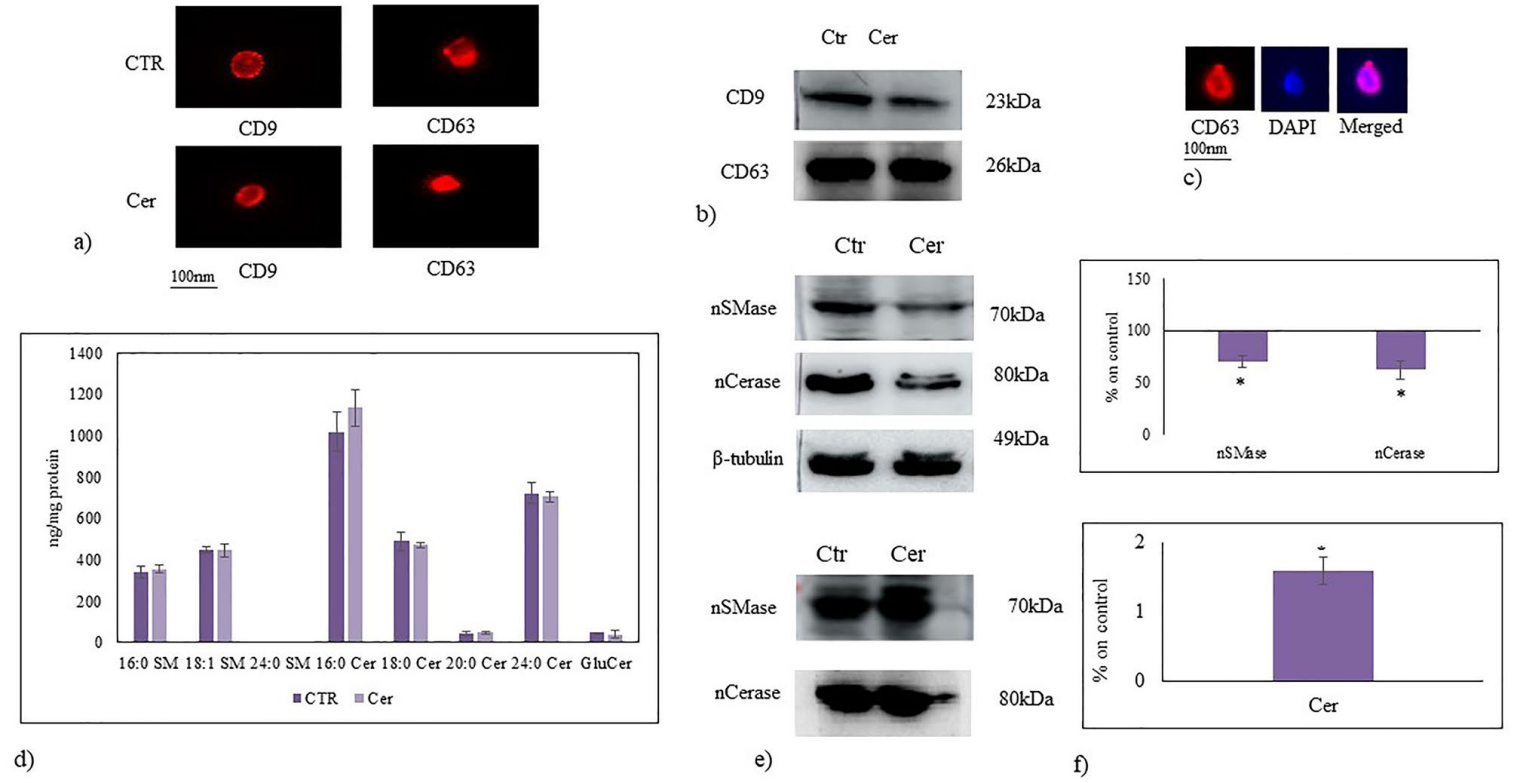
Characteristics of exosomes released from HN9.10e cells. Exosomes released from control cells (CTR) and ceramide treated cells (Cer). (**a**) Immunofluorescence of CD9 and CD63 as exosomal markers; (**b**) Western blotting of CD9 and CD63; (**c**) immunofluorescence of CD63, in red, counterstained with DAPI (in blue). Images were microscopically evaluated at 100 × magnification with immersion oil (DMRB Leika epi-fluorescent microscope equipped with a digital camera*)* and magnified with Photoshop programme; (**d**) UFLC MS/MS analysis of exosomes released under Ceramide treatment. Sphingomyelin (SM), ceramide (Cer) and glucosyl-ceramide (GluCer) species in exosomes released from control cells (CTR) and ceramide treated cells (Cer). Data are expressed as ng lipid/mg protein and represent the mean ± SD of three independent experiments performed in duplicate. Significance versus the control sample *p < 0.01; (**e**) Neutral sphingomyelinase and neutral ceramidase expression by western blotting: in top panel, cells cultured without (CTR) or with 100 nM Ceramide (Cer), β-tubulin was used as loading control and; in below panel, exosomes released from CTR and Cer cells. CD63 was used as loading control; (**f**) Quantification of area density by Chemidoc Imagequant LAS500 by specific IQ program, Data were expressed as mean ± SD of three independent experiments performed in duplicate. Significance versus the control sample, *p < 0.01. The values were first normalized with β-tubulin band (nuclei) or CD63 band (exosomes) and then calculated as percentage of CTR sample.

Based on the proposed role of Cer in embryonic hippocampal cell differentiation, we evaluated its effect on the possible release of exosomes from the cells which could justify the continuation and expansion of the differentiation process. For this, exosomes were isolated from culture medium and were tested for their specific markers CD9 and CD63^36^ by immunofluorescence (Fig. 2a) and immunoblotting analyses (Fig. 2b). The results showed the presence of both analyzed markers. Interestingly, counterstaining CD63 with DAPI, the positive reaction was highlighted (Fig. 2c).

Given the Sphs relevance for the structure/function of exosomes^37^ and our previous data showing the presence of SM, Cer and GluCer species in exosomes release from HN9.10e cells^23^, it became important to investigate whether ceramide C6 could somehow influence the composition of exosomal Sphs. Therefore, we analyzed SM, Cer species and GluCer with lipidomic analysis by using external calibrators and internal standards as reported in the “Method” section. The recovery of C12:0 SM as internal standard was 81% and 83% in the control and Cer treated samples, respectively. The recovery of C8:0 Cer was 85% and 86% in the control and Cer treated sample, respectively. The C6:0 Cer was absent in the control sample and it was equal to 12 ± 2 ng in exosomes isolated from the culture medium of HN9.10e Cer treated cells (1 × 10^6^ cells). Considering that 1 × 10^6^ cells were cultured in the presence of 150 ng Cer, 8% of the ceramide delivered to the cells was found in the exosomes. As shown in Fig. 2d, there is no significant difference of all the SM and Cer species and GluCer analyzed. Importantly, the treatment with Cer resulted in a significant decrease of nSMase and nCerase in the cells in parallel with their increase in exosomes (Fig. 2e,f).

### Exosomal miRNAs involvement as potential mediators of differentiating effects

**Figure 3:**
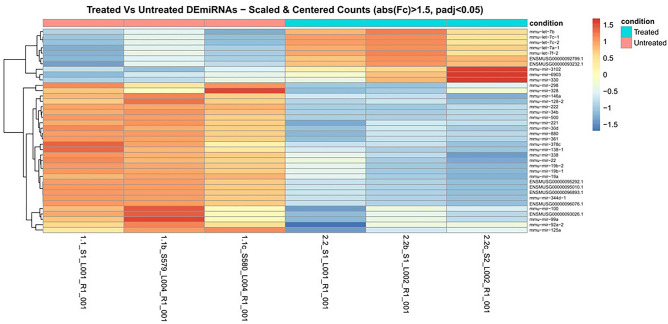
Exosomal miRNA profiling of HN9.10e cells treated with ceramide. Heatmap showing the statistically significant differentially expressed miRNAs in HN9.10e cells treated with ceramide. Globally, thirty-eight transcripts (n = 10 up-regulated, n = 28 down-regulated) were differentially expressed in the exosomes of HN9.10e cells treated with ceramide, compared to controls (abs(logFC) ≥ 0.6, q-value ≤ 0.05). Hierarchical clustering of transcripts and samples using the Euclidean distance and the complete agglomeration method; expression data was vst-transformed, scaled and centered. When not available, the miRBase accession number was replaced by the ENSEMBL GeneID. The heatmap was generated using the DESeq2 R package (v.1.0.12).

To determine the biological significance of exosomes released from the cells under Cer treatment, miRNAs were analyzed. Thus, exosomes were used to evaluate differentially expressed miRNAs (DE-miRNAs) upon Cer treatment. RNA-seq (abs(logFC) ≥ 0.6, q-value ≤ 0.05) analysis was performed to identify DEmiRNAs occurring between exosomes released from Cer treated cells and control cells (Table S1). By comparing both the distance matrix and the principal components analysis, we were able to confirm the good reproducibility of all biological replicas (Fig. S1). Among all the murine miRNAs profiled through the RNA-seq experiment (n = 1973), a total of 38 miRNAs resulted differentially expressed in a statistically significant manner, including 10 up-regulated and 28 down-regulated miRNAs (Fig. 3).

**Figure 4:**
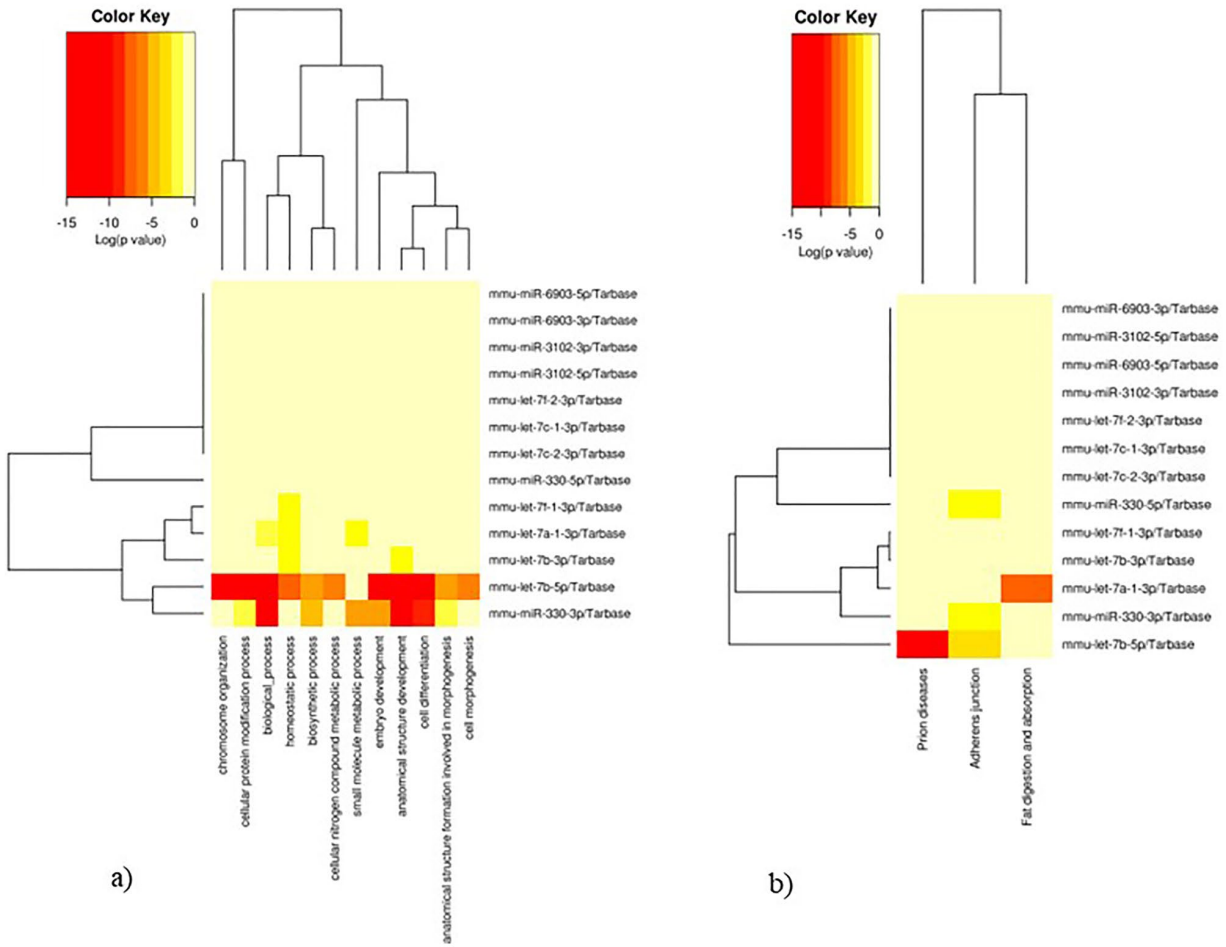
Functional enrichment analysis of differentially expressed miRNAs. Heatmaps showing the association of up-regulated miRNAs with: (**a**) “Gene Ontology-Biological Process” terms (“Categories Union” method) and, (**b**) with “KEGG Pathways” (“Pathways Union” method)^38,39^. The significance of each association is described by the color key. Heatmaps were generated using the DIANA-MirPath v.3 web server (last accessed July 22, 2022).

To investigate the role of DE-miRNAs, present in exosomes released from HN9.10e cells upon Cer treatment, in cellular processes, we assessed the enriched biological functions associated with their target genes. For each DE-miRNA (with the exception of Gm25927 that was not recognized) we defined the validated targets and common enriched functional terms of the 5p/-3p pairs, as described in the Methods section. Interestingly, both up as mmu-let-7f-1-3p, mmu-let-7a-1-3p, mmu-let-7b-3p, mmu-let-7b-5p, mmu-miR-330-3p (Fig. 4) and down-regulated (Fig. S2) miRNAs were associated with the regulation of lipid metabolism as well as cell differentiation and cell morphogenesis, indicating the existence of a complex regulatory network mediated by several DEmiRNAs affecting these biological processes.

**Figure 5:**
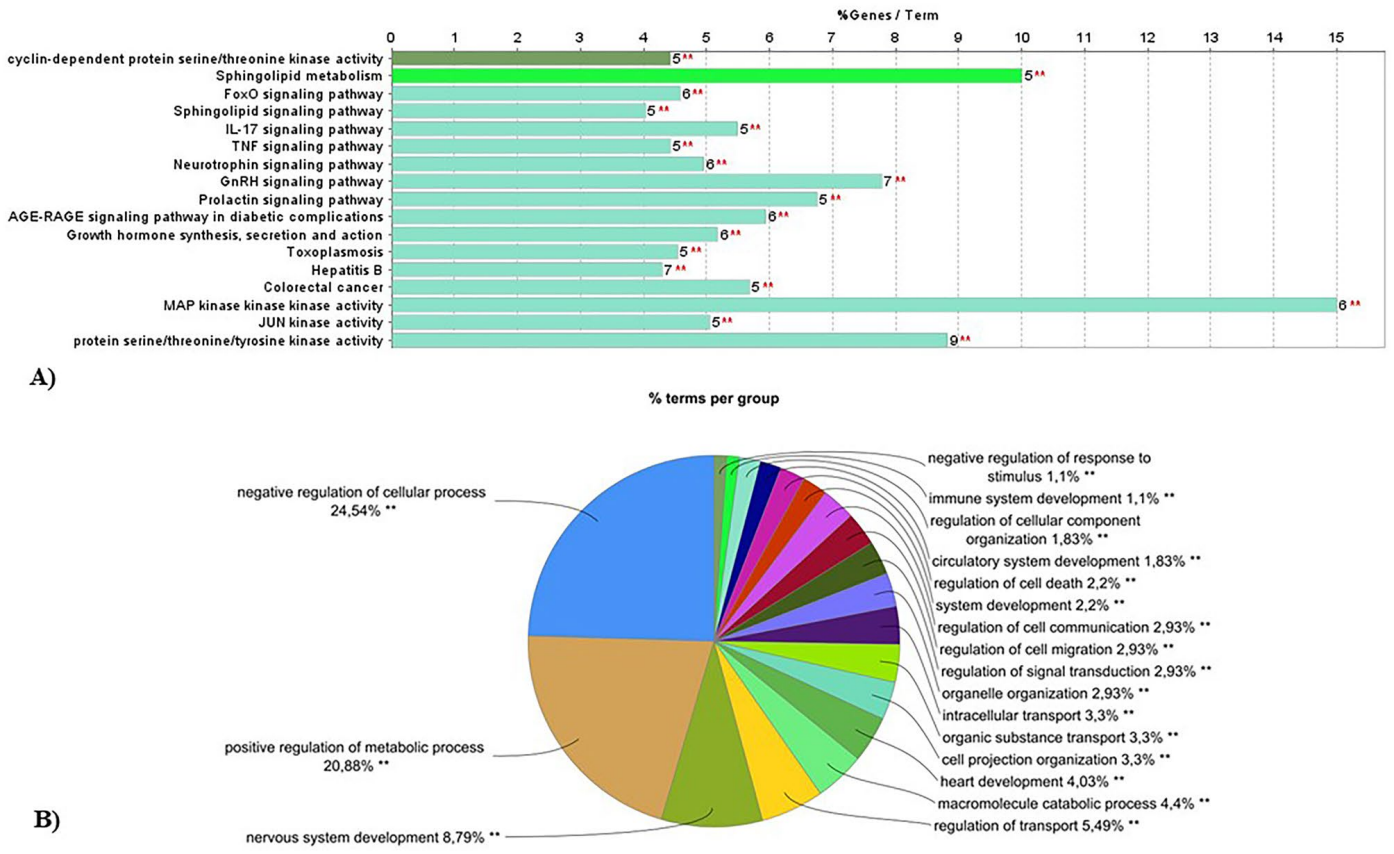
Functional enrichment analysis of mmu-let-7b-5p validated targets. The Cytoscape plugin ClueGO was used to identify the significantly enriched functional terms associated with a subset (n = 35) of in vitro validated targets (**A**), as well as the whole set of DIANA-TarBase v7.0 defined validated targets (Min Number of Genes n = 100, Min Percentage = 6.0) (**B**). Functionally enriched terms (Benjamini–Hochberg adjusted p ≤ 0.05) were identified querying the KEGG and the GO_BiologicalProcess databases^38,39^. The bar length (**A**) represents the percentage of genes associated with each enriched term that is found in the examines dataset, while the exact number of genes is indicated on the right of each bar. Pie chart colors (**B**) match the enriched functional clusters; the most significant terms were used as cluster representatives and identifiers comparison.

In regard to the results of the in vitro experiments described in the previous paragraphs, our attention focused on mmu-let-7b-5p, based on its ability to regulate thirty-five gene targets (Fig. 5) involved in processes including lipid transport, Sph metabolism and Sph-related stimulation of cellular functionalities. First, we investigated the biological processes associated with these selected genes using the ClueGO app, confirming their role in Sph metabolism and neuronal development (Fig. 5a). Afterwards, to have a broader, unbiased portrait of the mmu-let-7b-5p regulatory landscape, we repeated the analysis on all the previously defined DIANA-TarBase v7.0 murine validated targets (n = 1774), corroborating the effect on processes including regulation of transport, cell death, proteins/macromolecules metabolism and catabolism and, finally, system development with particular emphasis on nervous system development (Fig. 5b).

The effect of exosomes on HN9.10e cell differentiation was determined culturing the cells in the presence of increasing concentration of exosomes.

**Figure 6:**
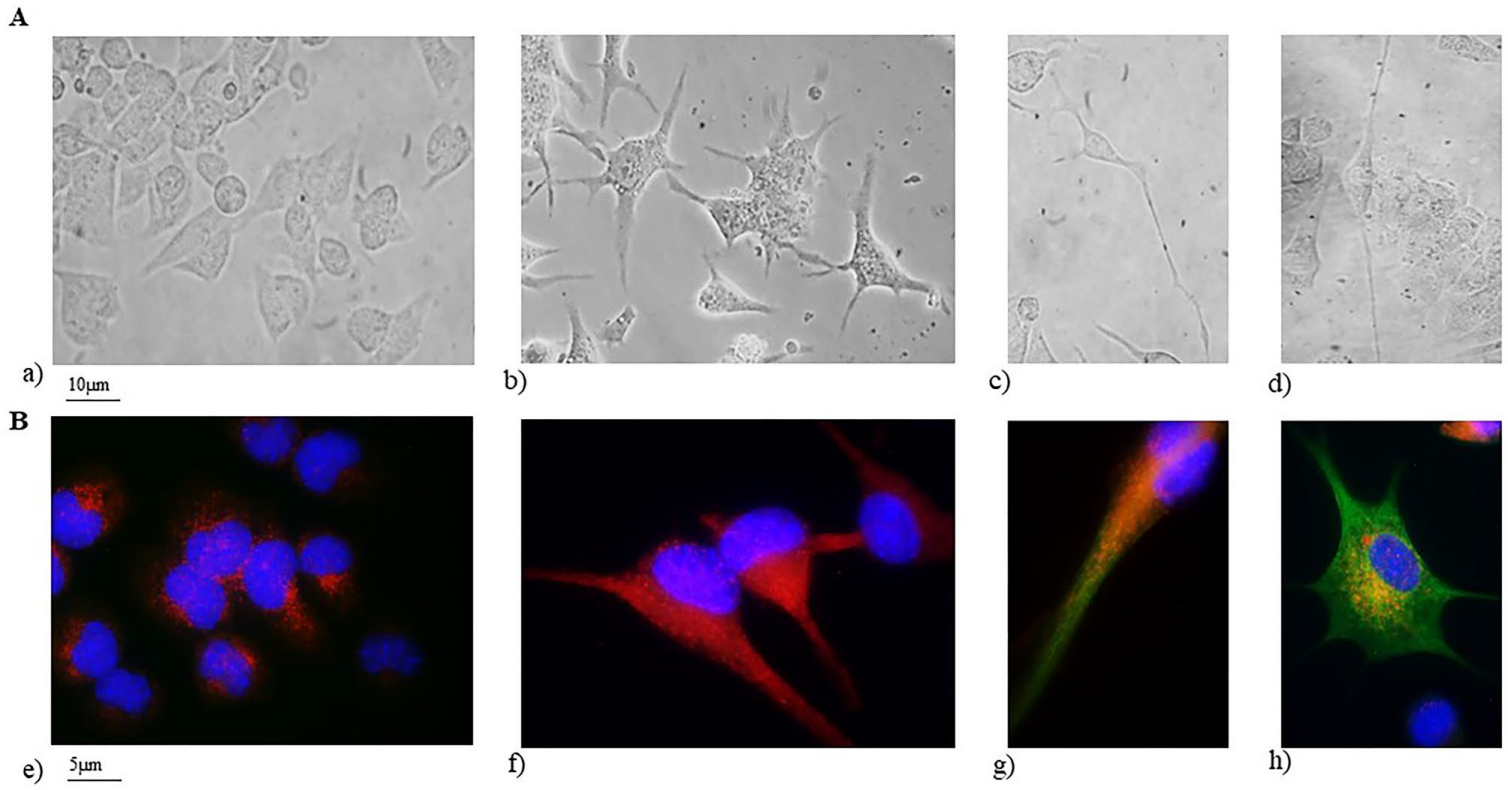
Effect of exosomes on HN9.10e cell differentiation. Cells were cultured for 96 h with exosomes released from Ceramide (Cer) treated cells and were analyzed as reported in the “Materials and methods”. (**A**) cell morphology of: (a) control and, (b–d) exosome treated sample. Observations were performed by using Olympus IX51 Inverted Microscope (40 × magnification) equipped with a digital camera; (**B**) immunofluorescence of GFAP and tubulin III. Observations were performed by using DMRB Leika epi-fluorescent microscope equipped with a digital camera (100 × magnification)*. *(e) control sample, the image shows low levels of GFAP expression (in red) and absence of βIII-tubulin expression (in green), characteristic of stem cells. The immunolabelling is counterstained with DAPI (in blue); (f) exosome treated sample, the image represents the merged signal of strong GFAP immunolabelling (in red) counterstained with DAPI (in blue), no βIII-tubulin signal (in green) is present, indicating cells differentiated towards the astrocytic phenotype; (g) exosome treated sample, the image shows the strong positive immunolabelling of βIII-tubulin (in green) with still weak stem cell GFAP labeling counterstained with DAPI (in blue). The cells are differentiating towards a unipolar cell neuronal phenotype; (h) as above reported (g) but the cells are differentiating towards a multipolar cell neuronal phenotype.

All concentrations used (protein from 200 to 300 µg/10^6^ cells) induced an increase in cell differentiation compared to untreated samples (data not shown). However, the optimal concentration corresponded to the concentration of exosomes released from cells under the Cer treatment (250 µg/10^6^ cells). As shown in Fig. 6A the cells appear completely modified in the volume and shape of the soma. Furthermore, neurites are formed differently in different cells, ranging from multipolar cells (Fig. 6Ab), to bipolar cells (Fig. 6Ac) and unipolar cells (Fig. 6Ad). To ascertain cell phenotype, the immunofluorescence to detect GFAP (astrocytes) and tubulin III (neurons) was performed. As shown in Fig. 6B a low level of GFAP in control cells was present equal to 15,400 ± 1800 fluorescence intensity (Fig. 6Be). Exosome treatment stimulated HN9.10e cell differentiation into both cell phenotypes, astrocytes as demonstrated by overexpression of GFAP (43,000 ± 100 fluorescence intensity, Fig.Bf) and neurons as demonstrated by overexpression of tubulin III (Fig. 6Bg,h).

## Discussion

In the present study, a novel role of Cer-induced exosome release has been identified. First, the data showed the expansion of the differentiation process of HN9.10e cells despite the single treatment with Cer at time 0. This finding suggested the possibility of a biochemical signal transmission via cell-to-cell communication.

The release of exosomes has been suggested as a potential mechanism to transfer active substances, as proteins, lipids, DNA, small nuclear RNAs, nucleolar RNAs, ribosomal RNAs, mitochondrial RNAs, miRNA, and small-molecule metabolites, from cells to body fluids and thus to other cells^36^. Previous studies suggested that exosomes play important roles in proliferation, angiogenesis, survival, and invasion of tumor cells^30^. However, their role in embryonic hippocampal cell differentiation has not been addressed previously. Therefore, we aimed to study the potential role of exosomes released from HN9.10e cells upon Cer stimulus on propagation of the differentiation signal in neighboring cells. The demonstration that the differentiation of HN9.10e cells upon Cer treatment increases in time in terms of cells with branching number and neurite length explains how Cer might be a key molecule in amplifying the response of HN9.10e cells to vitamin D3-induced differentiation, previously demonstrated^23^. In fact, previously reported findings by our group showed that vitamin D3 induced HN9.10e cell differentiation and released exosomes enriched in Cer content. The incubation of the cells with the same Cer concentration of that present in exosomes resulted in HN9.10e cell differentiation^23^. Here we describe the mechanism by which such differentiation stimulated by Cer occurs and its implication in propagation of the process. First, Cer itself stimulates the release of exosomes. Positive counterstaining with DAPI indicates the presence of nuclear material in exosomes, as reported by several authors (see a comprehensive review by Gurunathan et al.)^40^. We also demonstrated that the exosomal Sph composition is similar in control and Cer treated cells. Several previous reports have shown that SM and Cer are required for a proper structure/function of exosomes^41–43^. Mechanistically, the results suggested that Cer is able to stimuli the release of exosomes from cells without changing their structure. In support of this hypothesis, Verderio et al.^32^ demonstrated that Cer having a cone-shaped structure confers a spontaneous curvature to the membrane with release of exosomes. Interestingly, exosomes were released upon Cer treatment. The demonstration that after treatment of cells with Cer, nSMase decreases in whole cells and increases in exosomes suggests that exosomes can transport this enzyme to neighboring cells to facilitate the degradation of SM in them and resulting in Cer. This result connects the release of exosomes to the differentiative role of Cer. It can be speculated that the slight increase in nCerase content in exosomes released upon Cer treatment compared to control cell exosomes could be beneficial in the case of excess Cer production. To our knowledge, this is the first report identifying the information carried by the exosomes released from the hippocampus embryonic cells under the effect of Cer. Our results showed that out of a total of 1973 miRNAs, only 38 miRNAs are differentially expressed in a statistically significant manner, including 10 up-regulated and 28 down-regulated miRNAs. Both repressed and overexpressed miRNAs are relevant for biological modifications of nerve cells. Although exosomes contain different types of RNA miRNAs, long noncoding RNAs, circular RNAs, PIWI-interacting RNAs, and small interfering RNAs^24^, our attention has focused on miRNAs for their function as modulators of cell function including differentiation via regulation of gene expression^44^. Moreover, miRNA are thought to have diagnostic and prognostic values in neurodegenerative disease^45^. It will be important in the future to establish the different effect of each type of non-coding RNA on cell differentiation.

Fan et al.^46^ implicated the overexpression of miR-146a-5p, present in exosomes released from microglia cells of hippocampal dentate gyrus, in the suppression of neurogenesis. Interestingly, miR-146a-5p was down-regulated in our experimental model of exosomes, indicating the possible favorable effect in neurogenesis. Several miRNAs repressed in our experimental exosomes derived from Cer-treated cells were implicated in pathologies of the nervous system. Of note, serum miR22-3p was overexpressed in adolescents with attention deficit hyperactivity disorder^47^ and obsessive–compulsive disorder^48^. Moreover, serum exosomal miR-500-3p was upregulated in aging^49^. Additionally, sevoflurane exposure significantly decreased neuron cell proliferation with impairment of learning and memory via upregulation miR-19-3p^50^.

The overexpressed miRNAs deserve particular attention. Our analyses showed that overexpressed miRNAs (mmu-let-7f-1-3p, mmu-let-7a-1-3p, mmu-let-7b-3p, mmu-let-7b-5p, mmu-miR-330-3p) regulate genes encoding for proteins involved in biological, homeostatic, biosynthetic and small molecule metabolic processes, embryo development and cell differentiation, all phenomena that could be relevant for HN9.10e cell differentiation. Interestingly, among other overexpressed miRNAs, mmu-let-7b-5p turns out to be important for our study based on its ability to regulate thirty-five gene targets involved in many processes including Sph metabolism, Sph-related stimulation of cellular functionalities and neuronal development.

Together, these results have relevant implications for the role of exosomes released from embryonic hippocampal cells under Cer treatment in signal transfer for differentiation of neighboring cells both by regulating genes for the differentiation directly and via Sph metabolism.

In support, we cultured HN9.10e cells in the presence of exosomes to highlight their differentiative effect. We showed that the treatment induces the cells towards both the astrocytic and the neuronal phenotype. HN9.10e cells are embryonic cells that theoretically could differentiate into both phenotypes. The astrocytic phenotype was demonstrated with the overexpression of GFAP whereas the neuronal phenotype with the overexpression of βIII-tubulin. The permanence of a bland red color of positivity to GFAP in cells directed towards the neuronal phenotype indicates the persistence of the labeling of the starting embryonic cells which normally have a low expression of GFAP^51^. Thus, neurons are not yet fully differentiated but the direction towards the neuronal phenotype is now determined.

In summary, we describe the release of exosomes from HN9.10e cells under Cer treatment, and its biological significance in the differentiation of embryonic hippocampal cells towards astrocytes and neurons via miRNAs (Fig. S3). The study results are relevant to several research areas focused on cell–cell communication. The possibility that using other cell lines the results could be different cannot be excluded. Future studies may be aimed at consolidating this result in other cell lines. Notably, in the central nervous system Cer could be a target of innovative therapeutic strategies to regulate the release of exosomes to stimulate delayed brain development in the newborn. The research could be applied to adult hippocampal stem cells. In this way, the results could be useful to slow down and/or hinder the onset of neurodegenerative disorders and the consequent cognitive decline, including memory loss.

## Materials and methods

### Reagents

Dulbecco’s modified Eagle’s medium (DMEM), penicillin, streptomycin l-glutamine, trypsin-ethylenediaminetetraacetic acid disodium and tetra-sodium salt (EDTA) solution, fetal bovine serum (FBS) were from Microgem srl (Pozzuoli, NA, Italy); bovine serum albumin was from Thermo Fisher Scientific (Waltham, MA, USA); C6-Cer was obtained by Avanti Polar (Alabaster, AL, USA); anti-glial fibrillary acid protein (GFAP) and anti-βIII-tubulin antibodies were obtained from Dako, Agilent (Santa Clara, CA, USA); anti-SMase, anti-nCerase and anti βtubulin antibodies were from Abcam (Cambridge, UK); goat anti-rabbit secondary antibodies were from Sigma-Aldrich (St. Louis, MO, USA); anti-CD9 and anti-CD63 from Biorbyt (Cambridge, UK); anti-neurofilament protein (NF200) antibody and bond polymer refine detection were from NOVOCASTRA Laboratories, Ltd. (Newcastle, United Kingdom). For research involving biohazards, biological select agents and reagents, standard biosecurity safety procedures were carried out.

### Cell culture and treatments

Immortalized hippocampal neurons HN9.10e (kind gift of Kieran Breen, Ninewells Hospital, Dundee, UK) were grown in DMEM supplemented with 10% FBS, 2 mM l-glutamine, 100 IU/mL penicillin, 100 μg/mL streptomycin, and 2.5 μg/mL amphotericin B^22^. Cells were maintained at 37 °C in a saturating humidity atmosphere containing 95% air and 5% CO_2_. To study the effect of Cer on cell differentiation and exosome release, Cer was added to the culture medium at the 100 nmol/L concentration corresponding to the amount of Cer present in exosomes released under vitamin D3 treatment, as previously reported^23^. To investigate the effect of exosomes released under Cer treatment on cell differentiation, exosomes were added to the culture medium with increasing concentration of exosomes (protein concentration from 200 to 300 µg/10^6^ cells), considering that 1 × 10^6^ cells were able to release exosomes corresponding to 250 µg protein.

### Cell morphology

HN9.10e cells were cultured as above reported for 72 h and 96 h in absence or presence of 100 nM Cer. The observations were performed by using inverted microscopy EUROMEX FE 2935 (ED Amhem, Netherlands) equipped with a CMEX 5000 camera system (40 × magnification) and the morphometric analysis was performed by using ImageFocus software (EUROMEX, Arnhem, The Netherlands).

### Exosome isolation

Exosomes released in the culture medium from untreated HN9.10e cells (control sample) and Cer-treated HN9.10e cells (experimental sample) were isolated after 48 h of treatment. The supernatant was centrifuged at 2000×*g* × 30 min and filtered with 0.22 µm filter unit (MILLEX-GS from Millipore, Molsheim, France), in order to remove larger extracellular vesicles and apoptotic bodies, and concentrated with Pierce™ Protein Concentrators PES, 100 K MWCO from Thermo Scientific™. Then, the retained in the filter was used to isolate exosomes with “total exosome isolation” kit from Invitrogen-Thermo Fisher Scientific (Waltham, MA, USA) by following the instructions of the company.

### Electrophoresis and Western Blot analysis

Total protein concentration and protein expression analysis were performed as previously reported^22^. Briefly, 60 µg of protein were loaded on SDS–PAGE using 10% running gel. The transfer of protein was carried out onto nitrocellulose in 75 min^16^. The membranes were blocked for 60 min with 5% non-fat dry milk in PBS (pH 7.4) and incubated overnight at 4 °C with specific antibodies. The blots were treated with horseradish-conjugated secondary antibodies for 60 min. Band detection was performed using enhanced chemiluminescence kit from Amersham Pharmacia Biotech (Rainham, Essex, UK). A densitometric analysis was performed by Chemidoc Imagequant LAS500–Ge Healthcare-Life Science (Milano, Italy). Original WB images were reported as supplemental files (original WB cells and original WB exosomes). The tested antibodies were previously published in the same cell type^23,52^.

### Immunocytochemistry

HN9.10e were cultured for 48 h for immunocytochemical analysis. The cells were centrifuged at 1000×*g* for 10 min and the pellets were fixed in 10% neutral phosphate-buffered formaldehyde solution for 24 h. Then, the cytoinclusion technique by the Cellient® Automated Cell Block System (Hologic, Mississauga, ON, Canada) that rapidly creates a paraffin embedded cell block was used^53^. Bond Dewax solution was used to remove paraffin from sections before rehydration and immunostaining on the Bond automated system (Leica Biosystems Newcastle, Ltd., United Kingdom) as previously reported^53^. Immunostaining for neurofilament heavy protein (NF200) detection was performed by using specific antibody and Bond Polymer Refine Detection—Leica Biosystems (Newcastle, Ltd., United Kingdom). The observations were performed by using inverted microscopy EUROMEX FE 2935 (ED Amhem, Netherlands) equipped with a CMEX 5000 camera system (100 × magnification). The intensity of immunostaining was evaluated, as previously reported^49^.

### Immunofluorescence

Exosomes (125 µg in 200 µL) were set down on slides by centrifugation to 200 rpm on Shandon CytoSpin II Cytocentrifuge (Gmi, Inc. USA), immunolabelled with anti-CD9 and CD-63 primary antibodies diluted 1:40 for 1 h, washed and incubated with TRITC-conjugated anti rabbit IgG for 1 h. Diamidino-2-phenylindole (DAPI) was used to highlight nuclear material. Fluorescent analysis was performed on a DMRB Leika epi-fluorescent microscope equipped with a digital camera. Images were microscopically evaluated at 100 × magnification with immersion oil (DMRB Leika epi-fluorescent microscope equipped with a digital camera*)* and magnified with Photoshop program.

Cells cultured in the presence of exosomes were treated and incubated as previously reported^23^. Briefly, cells were incubated with anti-CD9, CD63, anti-GFAP or anti-tubulin III primary antibodies diluted 1:100 in 3% (*w/v*) BSA in PBS for 1 h, washed three times in 0.1% (*v/v*) Tween-20 in PBS and twice in PBS, incubated with tetramethylrhodamine isothiocyanate (TRITC)-conjugated anti-rabbit IgG for 1 h, diluted 1:50 in 3% (*w/v*) BSA in PBS and washed as above**.** The 4′,6-diamidino-2-phenylindole (DAPI) nuclear counterstain was used^54^. The samples were mounted in 80% (*w/v*) glycerol, containing 0.02% (*w/v*) NaN_3_ and *p*-phenylenediamine (1 mg/mL) in PBS to prevent fluorescence fading. The antibody incubations were done in a humid chamber at room temperature. Fluorescent analysis was performed on a DMRB Leika epi-fluorescent microscope equipped with a digital camera. The intensity of immunofluorescence was evaluated with Scion Image^55^.

### Ultrafast liquid chromatography–tandem mass spectrometry

Lipid extraction and analysis was performed as previously reported^2^. The pellets of the cells were suspended in Tris 10 mM, pH 7.4, and diluted with 1 mL methanol. Three milliliters of ultra-pure water and 3 mL methyl tert-butyl ether (MTBE) were added. Each sample was vortexed for 1 min and centrifuged at 3000×*g* for 5 min. The supernatant was recovered. The extraction with MTBE was repeated on the pellet and the supernatant was added to the first. The organic phase was dried under nitrogen flow and resuspended in 500 μL of methanol. The: 12:0 SM, 16:0 SM, 18:1 SM, 24:0 SM, sphingosine-1-phosphate, C18:0 sphinganine, C6:0 Cer, C8:0 Cer, C16:0 Cer, C18:0 Cer, C20:0 Cer, C24:0 Cer. C12:0 dihydroCer, arachidonoylglycerol (2AG), C16:0 glucosylceramide (GluCer) standards were dissolved in chloroform/methanol (9:1 vol/vol) at 10 μg/mL final concentration. The stock solutions were stored at − 20 °C. Working calibrators were prepared by diluting stock solutions with methanol to 500:1, 250:1, 100:1, and 50:1 ng/mL final concentrations. Twenty microliters of external standards or lipids extracted from serum with C12:0 SM and C8:0 Cer as internal standards diluted to 500 ng/mL (10 ng/mL final concentration) were injected after purification with specific nylon filters (0.2 μm). The analyses were carried out by using the Ultra Performance Liquid Chromatography system tandem mass spectrometer (Applied Biosystems, Italy). The lipid species were separated, identified, and analyzed as previously reported^23^. Liquid Chromatography system was Shimadzu Prominence UFLC, the pump was Shimadzu LC-20 AD, the detector was API 3200 linear triple quadrupole MS/MS, the injection valve was Valco valve, the autosampler was Schimadzu SR-20 AC HT, the column temperature stabilizer was Schimadzu CTO-20A. The samples were separated on a Phenomenex Kinetex phenyl-hexyl 100 A column (50 × 4.60-mm diameter, 2.6-μm particle diameter) with a precolumn security guard Phenomenex ULTRA phenyl-hexyl 4.6. Column temperature was set at 50 °C and the flow rate at 0.9 mL/min. Solvent A was 1% formic acid; solvent B was 100% isopropanol containing 0.1% formic acid. The run was performed for 3 min in 50% solvent B and then in a gradient to reach 100% solvent B in 5 min. The system needed to be reconditioned for 5 min with 50% solvent B before the next injection. The lipid species were identified by using positive turbo-ion spray (ESI) and modality multipole-reaction monitoring. Ion spray voltage was 5.4 kV, gas 1 was air, gas 2 was nitrogen, temperature was 650 °C, and the flow rate curtain gas was 40.5 L/h. The flow of the collision gas was maintained at 5.0 L/h. Data were acquired and processed using AnalystTM and Analyst 1.5 software in a Dell Precision T3400 personal computer with a Samsung ML-2851 MD graphical printer, as previously reported^23^.

### miRNA-seq

The exosomes-containing pellet was resuspended in 200ul of PBS buffer, quantified using the Exosome Quantitation Kit (System Biosciences) and stored at − 80 °C until use. Total RNA was extracted from 5 × 10^10^ exosomes using *mir*Vana miRNA Isolation kit (Life Technologies) and miRNA concentration was determined by Qubit microRNA assay kit (Invitrogen).miRNA expression profiling was performed with 15 ng of total RNA from exosomes released from HN9.10e cells under Cer treatment and from exosomes released from untreated HN9.10e cells as negative control. The experiment was performed in triplicates. RNA samples were quantified and quality tested by Agilent 2100 Bioanalyzer RNA assay (Agilent technologies, Santa Clara, CA) or Caliper (PerkinElmer, Waltham, MA). Sequencing libraries were prepared based on the QIAseq miRNA library kit (QIAGEN, Hilden, Germany), following the manufacturer’s instructions. Final libraries were checked with both Qubit 2.0 Fluorometer (Invitrogen, Carlsbad, CA) and Agilent Bioanalyzer DNA assay or Caliper (PerkinElmer, Waltham, MA). Quantified libraries were mixed at an equimolar ratio and sequenced on single-end 150 bp mode on NovaSeq 6000 (Illumina, San Diego, CA).

Raw sequencing data was processed for both format conversion and de-multiplexing using the Bcl2Fastq 2.20 version of the Illumina pipeline (https://support.illumina.com). The FastQC tool (ver 0.11.6) (https://www.bioinformatics.babraham.ac.uk/projects/fastqc/) was used to evaluate fastq files quality and the output was summarized with multiQC (ver1.4)^56^. Reads had very good quality and no correction was required. Cutadapt (ver 1.15)^57^ was used to remove adapter (AACTGTAGGCACCATCAAT (3′adapter)), primer (AGATCGGAAGAGCACACGTCTGAACTCCAGTCAC (partial RT and index primer)) and other types of unwanted sequences from the fastq files, applying the following parameters: -b AACTGTAGGCACCATCAAT-b AGATCGGAAGAGCACACGTCTGAACTCCAGTCAC-O 10-n 2-m 15. Alignment of reads was conducted with STAR (v2.5.3a)^58^, using the mouse genome assembly GRCm38 (mm10) with reference annotation; reads were assigned to a miRNA based on GENCODE annotations and via the STAR function “quantMode TranscriptomeSAM GeneCounts”. Differential expression (DE) analysis was performed using gene raw counts, within the R/Bioconductor DESeq2 package^59^; we estimated the dispersion parameter for each library using the biological group dispersion; abs(log2(fold change)) ≥ 0.6 (i.e. abs(FC) ≥ 1.5) was considered for differentially regulated genes; we adjusted the P value for multiple testing using the Benjamini–Hochberg correction with a false discovery rate (FDR) ≤ 0.05.

### miRNA targets definition and functional characterization

DE-miRNA murine validated targets were retrieved through the DIANA-MirPath v.3 web server^60^ Functional enrichment analysis was performed by querying the KEGG and Gene Ontology—Biological Process databases (p ≤ 0.05), applying the “genes union” and “pathways union” methods^61^. Additional information on selected DE-miRNA targets was retrieved.

### ClueGO functional enrichment analysis

The functional enrichment analysis of mmu-let-7b-5p selected target genes was performed using the Cytoscape^62^ app ClueGO^63^. Queried datasets: KEGG (update: May 13, 2021), GO_BiologicalProcess (update: May 13, 2021); Statistical Test Used: Enrichment/Depletion (Two-sided hypergeometric test); Correction Method Used: Benjamini-Hochberg; pvalue cutoff: 0.05; Min GO Level = 4; Max GO Level = 8; Min. Number of Genes = 5, Min Percentage = 4.0 (reduced target list); Min. Number of Genes = 100, Min Percentage = 6.0 (whole target list).

### Statistical analysis

Data were expressed as means ± SD of three independent experiments and significance was verified by Student’s *t*-test (experimental samples versus control sample).

## Supplementary Information

Supplementary Legends.

Supplementary Table S1.

Supplementary Figure S1.

Supplementary Figure S2.

Supplementary Figure S3.

Supplementary Information 6.

Supplementary Information 7.

## Data Availability

The datasets generated and/or analysed during the current study are available in the NCBI GEO repository, https://www.ncbi.nlm.nih.gov/geo/query/acc.cgi?&acc=GSE228052. Enter token qledumsytdkdbqt into the box.
