# Supplementary material for: Ceramide releases exosomes with a specific miRNA signature for cell differentiation
Source: Sci Rep. 2023 Jul 7;13:10993. doi: 10.1038/s41598-023-38011-1 (PMC10329022; doi:10.1038/s41598-023-38011-1)
Supplement: Supplementary file 1 — Supplementary Legends. [file 41598_2023_38011_MOESM1_ESM.docx]

**Table S1**. **Differentially expressed exosomal miRNAs in HN9.10 cells treated with ceramide**. miRNA-seq identified thirty-eight miRNAs that were either up-regulated (n=10, orange) or down-regulated (n=28, blue) in a statistically significant way in the exosomes of HN9.10 cells treated with ceramide, compared to controls (abs(logFC) ≥ 0.6, q-value ≤ 0.05). For each transcript, the fold-change in logarithmic scale, the significance (pvalue) and the corrected pvalue (qvalue) are shown.

**Figure S1. Principal component analysis (PCA) performed on the expression profiles of the exosomes released from ceramide-treated and control HN9.10e cells**. The two first principal components are plotted, explaining together 82% of the observed variance, and each experimental condition is highlighted by a different color. The samples form two separate and homogeneous clusters, confirming that the two experimental conditions represent distinct cell populations. The plot was generated using the DESeq2 R package (v.1.38.3)

**Figure S2. Functional enrichment analysis of differentially expressed miRNAs.** Heatmaps showing the association of up-regulated (**A, C**) and down-regulated (**B, D**) miRNAs with “Gene Ontology-Biological Process” terms (“Categories Union” method), and with “KEGG Pathways” (“Pathways Union” method). The significance of each association is described by the color key. The heatmap was generated using the DIANA-MirPath v.3 web server (last accessed July 22, 2022).

**Figure S3. Effect of exosomes released upon ceramide treatment on cell differentiation.** Ceramide acts on embryonic HN9.10e cells by stimulating the release of exosomes overexpressing miRNA that regulate genes for cell differentiation. As a consequence, neighboring cells change their soma shape and neurites appear, acquiring a mature cell phenotype.
