# Supplementary material for: Ceramide releases exosomes with a specific miRNA signature for cell differentiation
Source: Sci Rep. 2023 Jul 7;13:10993. doi: 10.1038/s41598-023-38011-1 (PMC10329022; doi:10.1038/s41598-023-38011-1)
Supplement: Supplementary file 2 — Supplementary Table S1. [file 41598_2023_38011_MOESM2_ESM.docx]

| **ENSMUSG** | **log2FoldChange** | **pvalue** | **qvalue** | **mirbase_id** |
| --- | --- | --- | --- | --- |
| ENSMUSG00000098606 | 1,52 | 8,49E-04 | 8,13E-03 | mmu-mir-6903 |
| ENSMUSG00000093296 | 1,00 | 2,49E-03 | 2,08E-02 | mmu-mir-3102 |
| ENSMUSG00000065543 | 0,86 | 5,01E-04 | 5,29E-03 | mmu-mir-330 |
| ENSMUSG00000093232 | 0,70 | 5,74E-06 | 1,39E-04 | Gm24111-201 (let-7 skeleton) |
| ENSMUSG00000065602 | 0,67 | 1,86E-05 | 3,19E-04 | mmu-let-7f-2 |
| ENSMUSG00000065557 | 0,67 | 6,82E-06 | 1,41E-04 | mmu-let-7c-1 |
| ENSMUSG00000065608 | 0,67 | 6,40E-06 | 1,40E-04 | mmu-let-7c-2 |
| ENSMUSG00000092799 | 0,65 | 1,62E-05 | 3,03E-04 | Gm25927 |
| ENSMUSG00000065421 | 0,63 | 1,83E-05 | 3,19E-04 | mmu-let-7a-1 |
| ENSMUSG00000065564 | 0,61 | 3,13E-05 | 4,61E-04 | mmu-let-7b |
| ENSMUSG00000093026 | -0,60 | 2,90E-04 | 3,15E-03 | Gm25301 (mir-378 skeleton) |
| ENSMUSG00000065601 | -0,64 | 1,44E-04 | 1,86E-03 | mmu-mir-146a |
| ENSMUSG00000065437 | -0,64 | 4,40E-06 | 1,13E-04 | mmu-mir-30d |
| ENSMUSG00000065613 | -0,66 | 1,32E-03 | 1,18E-02 | mmu-mir-92a-2 |
| ENSMUSG00000065529 | -0,66 | 2,80E-05 | 4,43E-04 | mmu-mir-22 |
| ENSMUSG00000093011 | -0,67 | 7,00E-04 | 6,86E-03 | mmu-mir-100 |
| ENSMUSG00000065600 | -0,70 | 1,51E-03 | 1,32E-02 | mmu-mir-338 |
| ENSMUSG00000065479 | -0,71 | 9,39E-06 | 1,84E-04 | mmu-mir-125a |
| ENSMUSG00000095292 | -0,73 | 3,24E-06 | 8,89E-05 | Mir344d-3 |
| ENSMUSG00000065414 | -0,74 | 2,01E-06 | 6,67E-05 | mmu-mir-138-1 |
| ENSMUSG00000092847 | -0,75 | 2,22E-06 | 6,67E-05 | mmu-mir-344d-1 |
| ENSMUSG00000095010 | -0,75 | 2,27E-06 | 6,67E-05 | Gm23490 (mir-344 skeleton) |
| ENSMUSG00000096893 | -0,75 | 2,27E-06 | 6,67E-05 | Mir344d-2 |
| ENSMUSG00000096076 | -0,75 | 2,19E-06 | 6,67E-05 | Gm23962 (mir-344 skeleton) |
| ENSMUSG00000065441 | -0,76 | 2,80E-05 | 4,43E-04 | mmu-mir-128-2 |
| ENSMUSG00000070130 | -0,76 | 2,48E-04 | 2,76E-03 | mmu-mir-328 |
| ENSMUSG00000065492 | -0,78 | 1,46E-07 | 7,54E-06 | mmu-mir-34b |
| ENSMUSG00000065410 | -0,85 | 1,70E-07 | 7,76E-06 | mmu-mir-298 |
| ENSMUSG00000065510 | -0,93 | 9,64E-11 | 6,62E-09 | mmu-mir-361 |
| ENSMUSG00000065530 | -0,94 | 5,24E-05 | 7,45E-04 | mmu-mir-99a |
| ENSMUSG00000065471 | -0,95 | 9,00E-08 | 5,30E-06 | mmu-mir-222 |
| ENSMUSG00000099036 | -1,03 | 1,23E-04 | 1,64E-03 | mmu-mir-378c |
| ENSMUSG00000078002 | -1,14 | 1,23E-11 | 1,01E-09 | mmu-mir-880 |
| ENSMUSG00000065422 | -1,18 | 1,28E-12 | 1,71E-10 | mmu-mir-221 |
| ENSMUSG00000070108 | -1,26 | 6,47E-06 | 1,40E-04 | mmu-mir-500 |
| ENSMUSG00000076256 | -1,35 | 4,11E-13 | 8,46E-11 | mmu-mir-19b-1 |
| ENSMUSG00000065473 | -1,35 | 3,57E-13 | 8,46E-11 | mmu-mir-19b-2 |
| ENSMUSG00000065416 | -1,36 | 1,66E-12 | 1,71E-10 | mmu-mir-19a |
