# Supplementary figures and images for: Ceramide releases exosomes with a specific miRNA signature for cell differentiation
Source: Sci Rep. 2023 Jul 7;13:10993. doi: 10.1038/s41598-023-38011-1 (PMC10329022; doi:10.1038/s41598-023-38011-1)

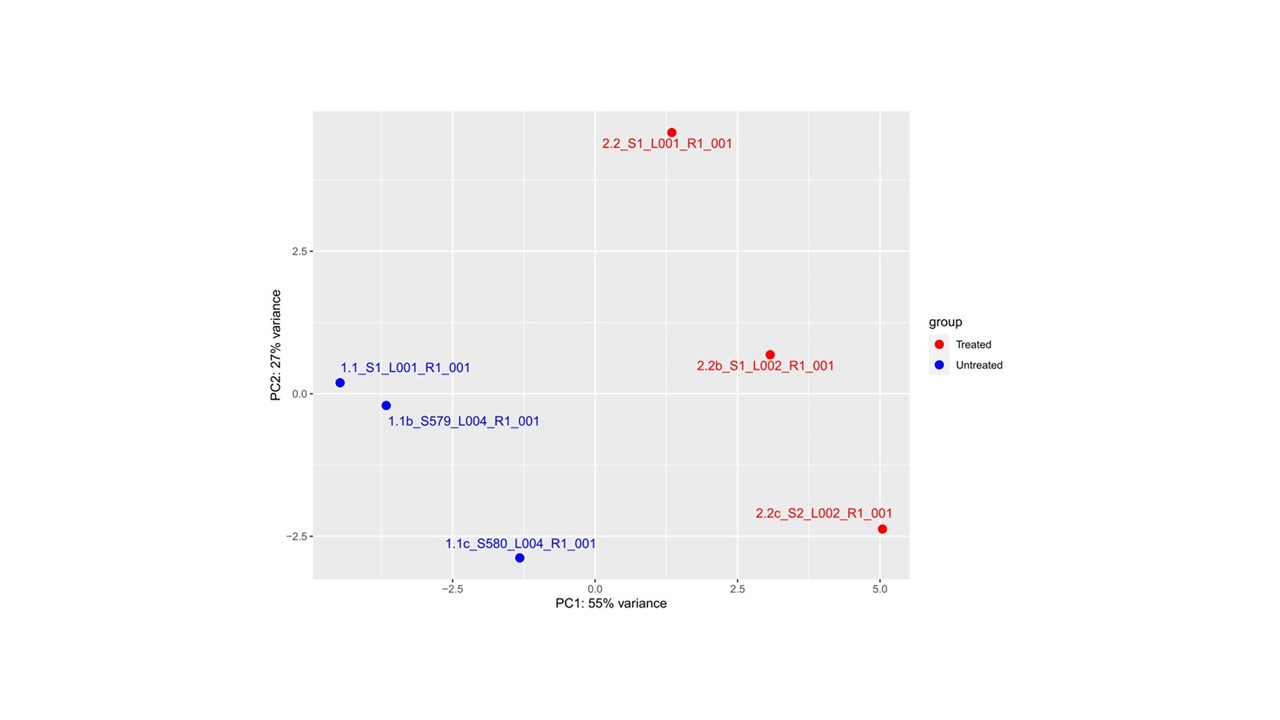

Supplement: Supplementary file 3 — Supplementary Figure S1. [file 41598_2023_38011_MOESM3_ESM.jpg]

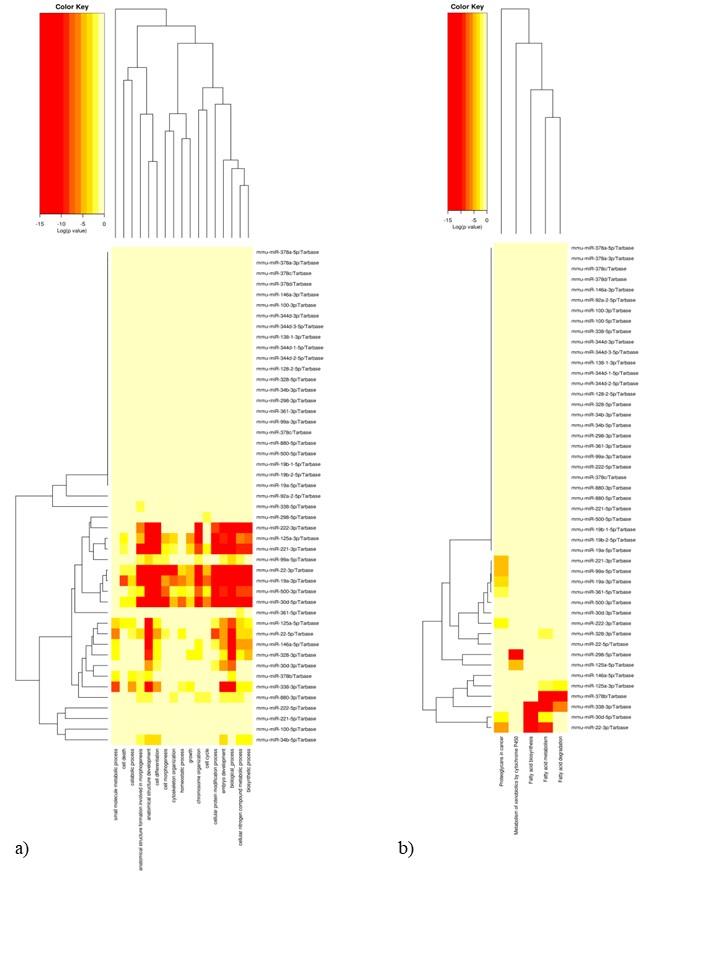

Supplement: Supplementary file 4 — Supplementary Figure S2. [file 41598_2023_38011_MOESM4_ESM.jpg]

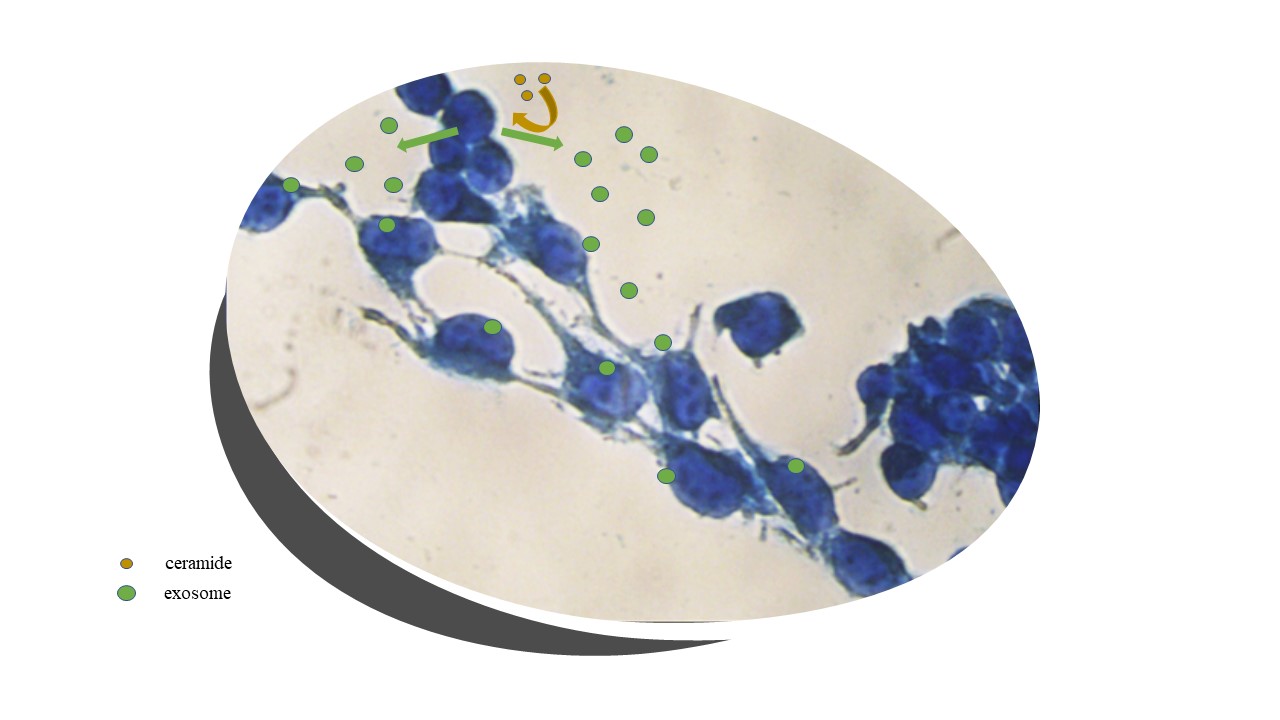

Supplement: Supplementary file 5 — Supplementary Figure S3. [file 41598_2023_38011_MOESM5_ESM.jpg]
