# Supplementary material for: Ceramide releases exosomes with a specific miRNA signature for cell differentiation
Source: Sci Rep. 2023 Jul 7;13:10993. doi: 10.1038/s41598-023-38011-1 (PMC10329022; doi:10.1038/s41598-023-38011-1)
Supplement: Supplementary file 6 — Supplementary Information 6. [file 41598_2023_38011_MOESM6_ESM.pdf]

Neutral sphingomyelinase
Abcam ab131330

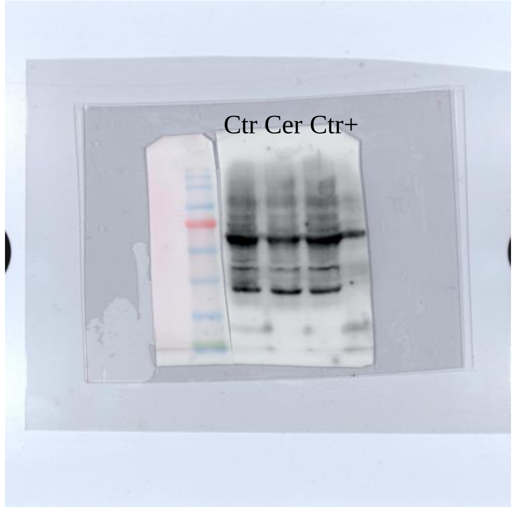

Neutral Ceramidase
Abcam ab252990

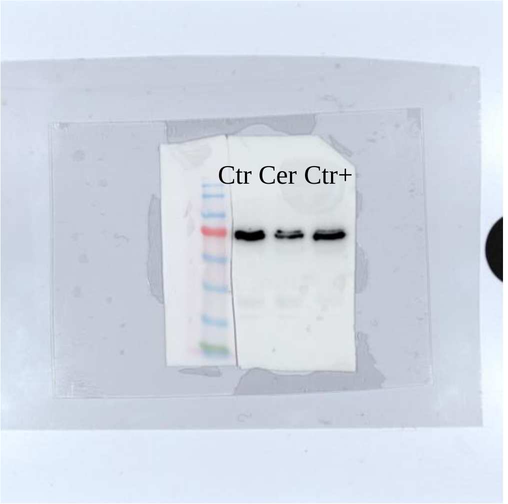

β Tubulina
abcam 131205

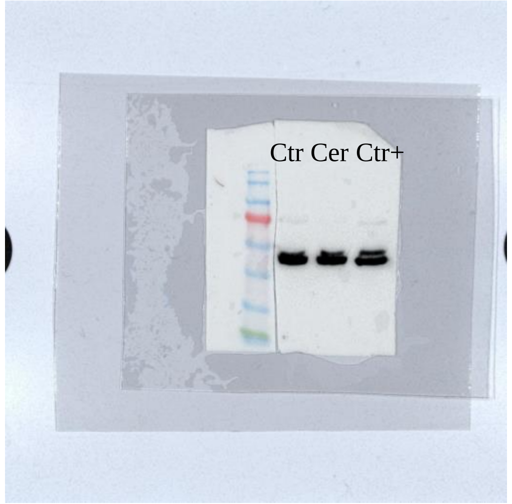

Original western blotting of the cells. The tested antibodies were previously published in the same cell type[23,48]. Ctr, control sample; Cer, ceramide trated sample; Ctr+, HaCaT cells used as positive control[58]
