# Supplementary material for: Ceramide releases exosomes with a specific miRNA signature for cell differentiation
Source: Sci Rep. 2023 Jul 7;13:10993. doi: 10.1038/s41598-023-38011-1 (PMC10329022; doi:10.1038/s41598-023-38011-1)
Supplement: Supplementary file 7 — Supplementary Information 7. [file 41598_2023_38011_MOESM7_ESM.pdf]

Neutral sphingomyelinase
Abcam (ab131330)

Ctr Cer

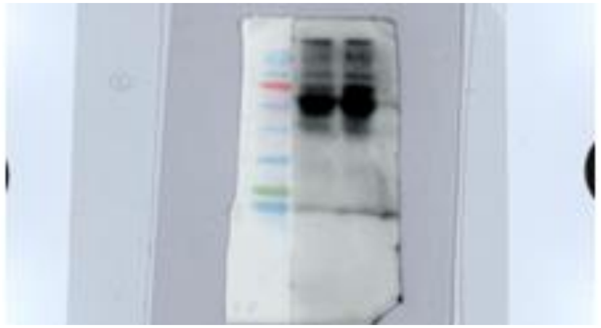

CD63
Biorbyt (orb11317)

Ctr Cer

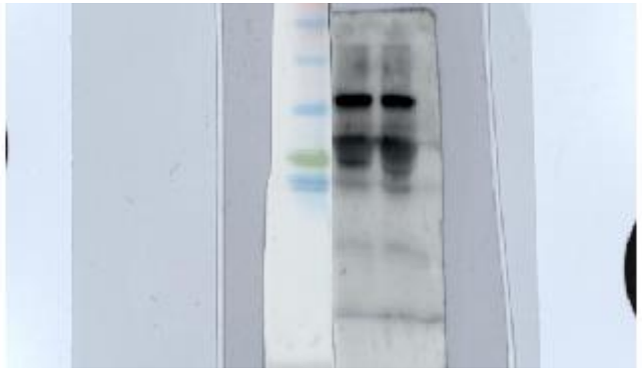

CD9
Biorbyt (orb235075)

Ctr Cer

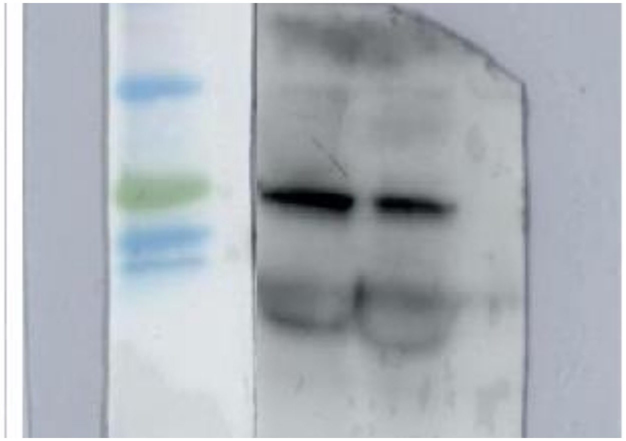

Neutral ceramidase
Abcam (ab252990)

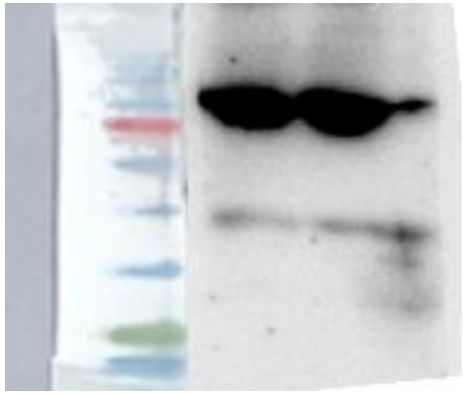

Original western blotting of the exosomes. The tested antibodies were previously published in the same cell type[23,48.] Ctr, control sample; Cer, ceramide trated sample.
